# Pre‐exposure prophylaxis initiation and adherence among Black men who have sex with men (MSM) in three US cities: results from the HPTN 073 study

**DOI:** 10.1002/jia2.25223

**Published:** 2019-02-15

**Authors:** Darrell P Wheeler, Sheldon D Fields, Geetha Beauchamp, Ying Q Chen, Lynda M Emel, Lisa Hightow‐Weidman, Christopher Hucks‐Ortiz, Irene Kuo, Jonathan Lucas, Manya Magnus, Kenneth H Mayer, LaRon E Nelson, Craig W Hendrix, Estelle Piwowar‐Manning, Steven Shoptaw, Phaedrea Watkins, C Chauncey Watson, Leo Wilton

**Affiliations:** ^1^ School of Social Welfare University at Albany – SUNY Albany NY USA; ^2^ School of Health Professions New York Institute of Technology New York NY USA; ^3^ Statistical Center for HIV/AIDS Research & Prevention (SCHARP) Vaccine and Infectious Disease Division (VIDD) Fred Hutchinson Cancer Research Center Seattle WA USA; ^4^ Statistical Center for HIV/AIDS Research & Prevention (SCHARP) Vaccine and Infectious Disease Division (VIDD) and Public Health Sciences Division Fred Hutchinson Cancer Research Center Seattle WA USA; ^5^ Division of Infectious Diseases University of North Carolina at Chapel Hill Chapel Hill NC USA; ^6^ John Wesley Community Health Institute, Inc. Commerce CA USA; ^7^ Department of Epidemiology and Biostatistics Milken Institute School of Public Health George Washington University Washington DC USA; ^8^ Science Facilitation Department FHI 360 Durham NC USA; ^9^ The Fenway Institute Fenway Health Boston MA USA; ^10^ Harvard Medical School Boston MA USA; ^11^ Beth Israel Deaconess Medical Center Boston MA USA; ^12^ School of Nursing University of Rochester Rochester NY USA; ^13^ Centre for Urban Health Solutions Li Ka Shing Knowledge Institute St. Michael's Hospital Toronto ON Canada; ^14^ Department of Medicine (Clinical Pharmacology) John Hopkins School of Medicine Baltimore MD USA; ^15^ Department of Pathology John Hopkins School of Medicine Baltimore MD USA; ^16^ Department of Family Medicine David Geffen School of Medicine University of California Los Angeles (UCLA) Los Angeles CA USA; ^17^ Gilead Sciences Foster City CA USA; ^18^ Department of Human Development State University of New York at BinghamtonBinghamton NY USA; ^19^ Faculty of Humanities University of Johannesburg Johannesburg South Africa; ^20^ Present address: CRS director for the GWU site and HPTN Black Caucus Chair

**Keywords:** HIV prevention, HIV disparities, MultiLevel interventions, PrEP initiation, PrEP adherence, client‐centered care coordination (C4)

## Abstract

**Introduction:**

Randomized clinical trials have demonstrated the efficacy of antiretroviral pre‐exposure prophylaxis (PrEP) in preventing HIV acquisition among men who have sex with men (MSM). However, limited research has examined initiation and adherence to PrEP among Black MSM (BMSM) in the United States (US) who are disproportionately represented among newly HIV infected and late to care individuals. This research reports on the HIV Prevention Trials Network 073 (HPTN 073) study aimed to examine PrEP initiation, utilization and adherence among Black MSM utilizing the theoretically principled, culturally informed and client‐centered care coordination (C4) model.

**Methods:**

The HPTN 073 study enrolled and followed 226 HIV‐uninfected Black MSM in three US cities (Los Angeles, CA; Washington DC; and Chapel Hill, NC) from February 2013 through September 2015. Study participants were offered once daily oral emtricitabine/tenofovir (FTC/TDF) PrEP combined with C4 and followed up for 52 weeks. Participants received HIV testing, risk reduction education and clinical monitoring.

**Results:**

Of the 226 men enrolled, 178 participants initiated PrEP (79%), and of these 64% demonstrated PrEP utilization at week 26 (mid‐point of the study) based on pharmacokinetic testing. Condomless anal sex with an HIV‐infected or unknown status casual male partner was statistically significantly associated with a greater likelihood of PrEP initiation (adjusted odds ratio (OR) 4.4, 95% confidence interval (CI) 1.7, 11.7). Greater age (≥25 vs. <25, OR 2.95, 95% CI 1.37 –6.37), perception of having enough money (OR 3.6, 95% CI 1.7 to 7.7) and knowledge of male partner taking PrEP before sex (OR 2.22, 95% CI 1.03 to 4.79) were statistically significantly associated with increased likelihood of PrEP adherence at week 26. Annualized HIV incidence was 2.9 (95% CI 1.2 to 7.9) among those who initiated PrEP, compared to 7.7 (95% CI 2.5 to 24.1) among those who did not initiate PrEP (*p* = 0.18).

**Conclusions:**

Results suggest a high level of PrEP initiation among at‐risk Black MSM, a group historically characterized as hard to reach. The data support the importance of addressing contextual factors that affect PrEP initiation and adherence, and of additional research on the ultimate benefit of PrEP in HIV prevention among Black MSM.

## 1 Introduction

In 2016, Black men who have sex with men (BMSM) constituted the highest proportion (38%) of new HIV infections diagnosed in the US as compared to Latino (28%) and White (28%) MSM 1, 2, 3, 4, 5. Based on current rates of HIV diagnoses epidemiological data suggest Black US MSM have a 50% probability of acquiring HIV in their lifetime with young Black men (under 25) being at significant risk within this population 6. Identifying effective interventions to reduce HIV burden among Black MSM is a priority in the US National HIV Strategy 7.

A constellation of factors contribute to increased HIV infections among Black MSM, including higher levels of unrecognized HIV and sexually transmitted infections (STI), and delayed initiation of antiretroviral therapy 3, 5, 8, 9, 10, 11, 12. Structural factors, such as lower income, un‐/under‐employment, educational inequalities, inadequate access to healthcare and treatment, incarceration, stigma and discrimination also influence the incidence and prevalence of HIV among Black MSM 13, 14, 15, 16, 17, 18, 19. Research also documents inadequate incorporation of Black researchers in conducting HIV prevention and clinical studies focused on Black communities. This lack of representation may influence the design and interpretation of studies 20.

Along with behavioural interventions 21, 22, 23, 24, 25, 26, early HIV diagnosis and linkage to care and treatment 27, 28, 29, use of antiretroviral drugs as pre‐exposure prophylaxis (PrEP) is critical for the prevention of HIV acquisition 30, 31, 32, 33, 34, 35, 36. Clinical studies have demonstrated the efficacy of oral daily PrEP along with counselling in preventing HIV acquisition in MSM 31, 32, 37. Mathematical modelling indicates that approximately 20% to 25% of new HIV infections among MSM globally could be reduced with expanded access to PrEP 38, 39.

Limited research is available on the initiation of and adherence to PrEP among Black MSM in the US 40, 41, 42, 43, 44, 45. The importance of developing effective interventions and methods for delivering these interventions is essential to reducing the disproportionate HIV burden among BMSM in the United States inclusive of health provider biases 21, 30, 40.

Evidence‐based research is needed to address this vital public health priority, including culturally sensitive care and support services that address the structural barriers 40, 41, 42, 43, 44, 45. We report results from an open‐label antiretroviral PrEP demonstration project, HIV Prevention Trials Network 073 (HPTN 073) that examined the uptake/initiation and adherence with daily oral co‐formulated emtricitabine and tenofovir disoproxil fumarate (FTC/TDF) among Black MSM in three US cities.

A theoretically principled and culturally informed intervention, client‐centred care coordination (C4), was developed for use in this study to support participants’ PrEP understanding, initiation and adherence 34, 35, 36, 37.

## 2 Methods

### 2.1 Ethics statement

The HPTN 073 Study protocol and all related materials were reviewed and approved by institutional review boards of University of California at Los Angeles, The University of North Carolina at Chapel Hill and George Washington University. All study participants provided written informed consent and completed an informed consent assessment to ensure a thorough understanding of the study prior to enrolment. Ongoing informed consent was assessed and confirmed at each visit.

### 2.2 Study design and participants

This non‐randomized open‐label PrEP study was conducted among MSM who self‐identified as Black in three US cities. Enrolment eligibility criteria included: (1) being 18 years and older; (2) serologically confirmed HIV‐uninfected; (3) biologically male at birth; (4) self‐report at least one of the following: condomless receptive or insertive anal intercourse with a male partner, having any anal intercourse with more than three male sex partners; having exchanged any anal sex with a male partner for money gifts, shelter or drugs; having any anal intercourse with a male partner while using drugs or alcohol; diagnosed with an STI and having a male sex partner in the past six months; (5) willingness to provide locator information for study follow‐up; and (6) meeting clinical safety criteria related to PrEP eligibility including urine dipstick negative or trace for protein and glucose, haemoglobin >11 g/dL, and being HBV‐uninfected. Potential participants were excluded if they reported using antiretroviral drugs in the 60 days prior to enrolment or if they were unable to provide informed consent.

### 2.3 Recruitment and follow‐up

Recruitment of Black MSM participants occurred between February 2013 and September 2014. Study sites were Washington, The District of Columbia; Los Angeles, California; and Chapel Hill, North Carolina. Each site participated in community outreach and cultural competency training developed and facilitated by the HPTN Black Caucus 46 to ensure consistency in outreach, recruitment and implementation efforts while respecting site‐specific approaches to local recruitment methods. Recruitment strategies included peer referral, venue‐based sampling, local media and word‐of‐mouth often conveyed by local health providers or others engaged with PrEP and/or Black MSM communities. Study participants were followed up for a 52‐week period.

### 2.4 Interviewer administered questions

Trained interviewers collected sociodemographic (e.g. age, sexual identity) participant data 5, 20. Perception of insufficient income was assessed by asking participants whether they had enough money for household expenses, such as rent, food and/or utilities in the six months prior to assessment and was coded as a dichotomous variable: (1) Yes=once in a while, fairly often or very often and (2) No=never.

### 2.5 ACASI administered questions

Participants completed an audio‐computer assisted self‐interview (ACASI) at enrolment and study visits at weeks 4, 8, 13, 26, 39 and 52, including items described below. Sexual behaviour items adapted from previous research 22 with Black MSM included primary male partners (defined as “someone who you would describe as your boyfriend, lover, life partner, or someone you may have lived with or saw a lot, or to whom you felt a special emotional commitment”), casual male partners (defined as someone who you had sex with casually or would describe as a one‐night stand, sex buddy, anonymous sex partner, or another male sex partner who was not your primary or main partner), cisgender female partners, and exchange sex partners, and insertive and receptive anal intercourse acts with primary and casual male partners during the three months prior to assessment. Anal intercourse items (insertive and receptive) were stratified by the HIV serostatus of male sexual partners (HIV‐infected, HIV non‐infected and HIV status unknown). Items related to female cisgender sex partners included vaginal and anal sex (with and without condoms) during the three months prior to assessment.

Substance use items asked about the self‐reported frequency of (never; once; one to two days a month; three to four days a month; one to two days a week; three to four days a week; daily) alcohol and drug use within the three months prior to assessment. Substances assessed included alcohol, marijuana, cocaine (crack and powder), methamphetamine, heroin, non‐prescribed painkillers or anti‐anxiety medications, non‐prescribed erectile promotion drugs, inhaled nitrates (poppers), anabolic steroids and female sex hormones. Based on prior studies of substance use with Black MSM 47, 48, 49, 50, a binary variable (Yes/No) was created to measure substance use.

#### Depressive symptoms

A brief version of the Center for Epidemiologic Depression (CESD) Scale (CESD‐10) was used to assess depressive symptoms 51, 52 (alpha reliability coefficient = 0.89). A sum of the scores was computed using a cutoff score of ≥10 to categorize participants as being positive for depressive symptoms.

#### Incarceration

Based on research with Black MSM 18 incarceration was defined as having ever spent one or more nights in a jail, detention facility or prison.

#### PrEP‐related knowledge

Two items measured aspects of PrEP knowledge: (1) knowledge that an HIV‐negative partner was taking PrEP before having sex and (2) knowledge that PrEP could be used to prevent HIV infection 5.

### 2.6 Testing

All participants were asked to provide specimens for HIV, STI and measuring PrEP drug levels at study visits. HIV testing was performed at each study visit using US Food and Drug Administration (FDA)‐approved assays, including at least one HIV rapid test and at least one‐fourth‐generation HIV test. Serologic tests for syphilis were performed using local site‐specific testing algorithms. Testing for *Chlamydia trachomatis* (CT) and *Neisseria gonorrhoeae* (NG) were performed for urine and rectal swab samples using the Aptima Combo 2 Assay for CT/NG assay at the local laboratories.

For all participants, creatinine clearance was measured at the screening visit, and at the 4 and 13‐week postenrolment visits and quarterly thereafter if the participant‐initiated PrEP 53, 54. Urine dipstick for protein and glucose was also performed at screening and quarterly after enrolment. Participants with laboratory abnormalities had their findings and clinical care on protocol managed by study clinicians. When indicated, participants were referred to primary care physicians in the community for ongoing or non‐study‐related clinical assistance.

### 2.7 Client‐centred care coordination (C4) intervention and PrEP

The C4 intervention integrates an evidenced‐based public health strategy comprehensive risk counselling and services (CRCS) with a self‐determination theory (SDT)‐based approach to counselling and client engagement 55, 56. The CRCS combines HIV risk reduction counselling with case management activities to address potential barriers to HIV prevention goal attainment 57. Both provided conceptual and explanatory bases for C4 activities including behavioural counselling, care coordination, referrals and follow‐up care. The use of SDT as a foundation for the C4 intervention to facilitate PrEP initiation and adherence was supported by prior research indicating that SDT was associated with increased recent HIV prevention behaviours (condom use for anal sex) among MSM 58 and antiretroviral medication adherence 56. Moreover, the use of this theoretical foundation was supported by a well‐established research literature on the efficacy of SDT‐based approaches in promoting behavioural risk‐reduction and treatment adherence in other health domains 59, 60, 61, 62. Critical to C4 success was the training and ability of counsellors in assisting participants to address complex health and social realities of BMSM (present and historical) and demonstrating connection and caring for the life beyond adherence to a medication regimen 55.

### 2.8 C4 encounters

All participants received an initial C4 session in which they identified HIV prevention goals in the context of possible PrEP initiation and adherence. C4 counselling was offered at each study visit, and participants could decline the counselling if not desired. Case report forms documented C4 sessions, and the number of encounters was calculated as a continuous variable.

### 2.9 PrEP

Participants were offered and could initiate their study PrEP regimen at any time between enrolment and week 48 of the study period. Men seeking to initiate PrEP after this period were referred to community providers. Participants were under no obligation to initiate PrEP and could start and stop taking it at any time during the study. The C4 model included transitioning to community‐based clinical management of PrEP after participants exited the study at week 52.

No C4 follow‐up support was provided after participants exited the study.

### 2.10 HIV testing

The HPTN Laboratory Center (LC) performed Quality Assurance testing using the Abbott Architect HIV 1/2 Combo test. Incident HIV infections were confirmed at the HPTN LC using a panel of assays that included the Bio‐Rad GS Combo Ag/Ab EIA test and the Bio‐Rad MultiSpot HIV‐1/HIV‐2 Rapid test. Qualitative HIV RNA testing was performed using the APTIMA HIV‐1 RNA Qualitative Assay to determine if the participant had acute HIV infection at the visit prior to HIV seroconversion. Viral load and CD4 cell count testing were performed at study sites for participants who acquired HIV infection during the study.

### 2.11 Safety monitoring

Assessment for adverse events (AEs) was performed at follow‐up visits for all study participants, regardless of whether they were or were not on PrEP. Clinically diagnosed STIs and all conditions that resulted in a clinical hold or permanent discontinuation of the study PrEP regimen were reported as AEs. Conditions that caused the study PrEP to be held or permanently discontinued included regimen‐related toxicity or abnormal laboratory values; investigator decision; a reactive HIV test or concern about acute HIV infection; participant decision to stop study drug or request “drug holiday”; missed visits; or participant hospitalization. AEs were graded using the NIH DAIDS AE Grading Table, Version 1.0, December 2004 62.

### 2.12 Study outcomes: PrEP initiation and adherence

The main outcomes for this non‐randomized acceptability and feasibility study were PrEP initiation and adherence, measured as the percentage of men who initiated study PrEP at any point from enrolment to week 48 of the follow‐up period. PrEP initiation was defined as the date the participant took the first dose, by self‐report, and adherence was determined by pharmacological testing of two types of participant specimens: plasma and peripheral blood mononuclear cells (PBMCs) collected at week 26 (midpoint of the study) 32. PrEP adherence was defined as those who met the 90% sensitivity threshold for ≥4 doses of FTC/TDF per week from any of the two samples types (Plasma and PBMC) related to tenofovir (TFV) and FTC measurements: ≥4.2 ng/mL for TFV and ≥4.6 ng/mL for FTC in plasma; 9.9 fmol/10^6^ for TFV diphosphate and 0.4 fmol/10^6^ for FTC triphosphate in PBMCs. These measures of adherence for plasma and PBMC samples were established by the directly observed daily dosing study, HPTN 066 63.

### 2.13 Statistical methods

Baseline characteristics, socio‐demographics, sexual behaviour, incarceration, substance use and C4 encounters were summarized using measures of central tendency. PrEP initiation, and adherence were estimated using proportions. Correlates of PrEP initiation and PrEP adherence at the week 26 study visit for those who had initiated PrEP were examined separately using univariate and multivariate adjusted logistic regression models. Correlates that were identified in univariate logistic regression models with *p *<* *0.05 were further selected for inclusion in backward stepwise multivariate logistic regression models. The cutoff of *p* < 0.05 in the multivariate logistic regression models was considered statistically significant. In all models, study site was included as a covariate to account for heterogeneity among the sites. HIV incidence rates were calculated using person‐year analysis. For those who seroconverted, person‐year is calculated from the enrolment date to first HIV‐positive test result date, and for those who remained HIV negative, the person year is calculated from enrolment date to the last test result date. All statistical analyses were performed using SAS version 9.4.

## 3 Results

### 3.1 Study recruitment and retention

**Figure 1:**
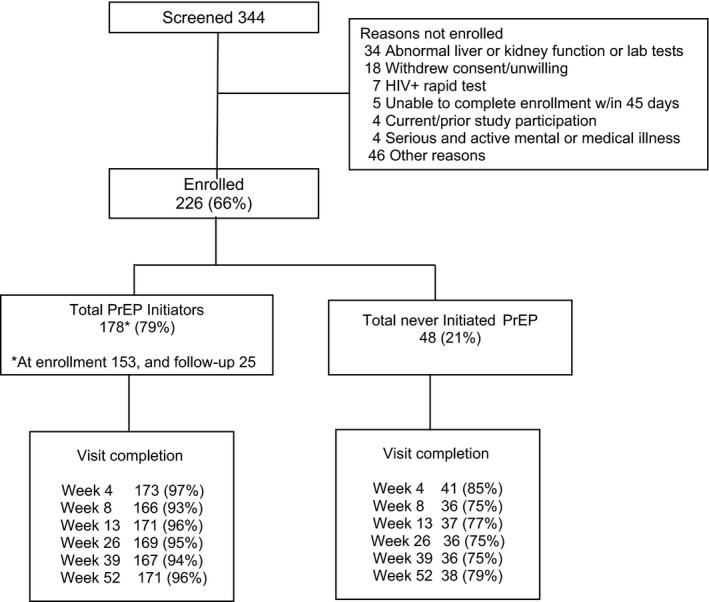
Study CONSORT diagram

Of the 344 individuals screened, 226 eligible participants enrolled (66%) in the study. Abnormal liver or kidney laboratory tests were the most common reasons for ineligibility (Figure 1). Study retention was 92% of enrolled participants completing the week 52 follow‐up visit.

### 3.2 Participant characteristics

Of the 226 participants enrolled, 86% self‐identified as Black or African American only, while the remaining included Afro‐Caribbean, African and Afro‐Latino. Median age was 26 (IQR: 23 to 32) with 40% being 25 years old or younger. One‐quarter (25%) had a high school diploma or less; more than one‐third (38%) worked full‐time, and 27% were unemployed; 48% reported an annual income of less than $20,000. More than half (69%) had health insurance. Nearly three‐quarters identified as gay (73%) and 20% identified as bisexual. Most participants were single (83%). Of the 39 individuals who reported being in relationships, 36 (92%) had male partners (Table 1).

**Table 1:** Baseline characteristics of Black men who have sex with men (MSM) enrolled in HIV prevention trials network study 073; N = 226

	GWU^a^ (N = 75)	UCLA^a^ (N = 76)	UNC AIDS^a^ (N = 75)	Total^a^ (N = 226)
PrEP initiated^b^	80% (60)	67% (67)	89% (67)	79% (178)
Demographic characteristics				
African American^c^	84% (63)	82% (62)	93% (70)	86% (195)
Age ≥25 years	55% (41)	70% (53)	55% (41)	60% (135)
Median (IQR)	28 (23 to 29)	32 (24 to 39)	28 (22 to 31)	26 (23 to 32)
Highest education level attained				
HS or less	17% (13)	34% (26)	23% (17)	25% (56)
Some college or vocational school	40% (30)	36% (27)	48% (36)	41% (93)
Two‐year college or greater	43% (32)	30% (23)	29% (22)	34% (77)
Employment status				
Unemployed, disabled or other	20% (15)	38% (29)	23% (17)	27% (61)
Part time or self‐employed	29% (22)	37% (28)	40% (30)	35% (80)
Full time	51% (38)	25% (19)	37% (28)	38% (85)
Annual income				
No response	4% (3)	0% (0)	0% (0)	1% (3)
<$20K	23% (17)	66% (50)	55% (41)	48% (108)
$20K to $40K	21% (16)	21% (16)	31% (23)	25% (55)
≥$40K	52% (39)	13% (10)	15% (11)	27% (60)
Have enough money	65% (49)	45% (34)	68% (51)	59% (134)
Health insurance (current)	80% (60)	63% (48)	63% (47)	69% (155)
Gay	80% (60)	74% (56)	67% (50)	73% (166)
Bisexual	16% (12)	21% (16)	23% (17)	20% (45)
Married/significant other	25% (19)	13% (10)	13% (10)	17% (39)
Ever been incarcerated	26% (19)	49% (37)	18% (13)	31% (69)
Depressive symptoms^d^	25% (19)	33% (25)	29% (22)	29% (66)
Substance use in the last three months				
Any alcohol use	89% (67)	83% (63)	92% (69)	88% (199)
Any marijuana use	53% (40)	57% (43)	36% (27)	49% (110)
Any other drug use	85% (64)	72% (55)	92% (69)	83% (188)
Any poly drug use	7% (5)	18% (14)	11% (8)	12% (27)
Sexual behaviours/characteristics				
Age at first sexual encounter, median (Q1, Q3)	14 (10,16)	13 (8,15)	14 (12,17)	14 (10,16)
Any baseline sexually transmitted infection	16% (12)	9% (7)	17% (13)	14% (32)
Sexual behaviours in the last three months				
Number of male partners, median (IQR)	2 (1,4)	3 (1,5)	3 (1,4)	3 (1,4)
Sex with a male partner	93% (70)	93% (71)	92% (69)	93% (210)
Had a primary male partner	35% (26)	38% (29)	25% (19)	33% (74)
Any condomless anal sex with HIV positive or unknown primary male partner	13% (10)	9% (7)	9% (7)	11% (24)
Had a casual male sex partners	64% (48)	71% (54)	79% (59)	71% (161)
Any condomless anal sex with HIV positive or unknown casual male partner	28% (21)	37% (28)	37% (28)	34% (77)
Had a female sex partner	8% (6)	13% (10)	15% (11)	12% (27)
Any condomless anal sex with female partner	7% (5)	11% (8)	4% (3)	7% (16)
Exchanged sex for money	11% (8)	16% (12)	9% (7)	12% (27)

GWU, George Washington University; IQR, interquartile range; UCLA, University of California at Los Angeles; UNC AIDS, University of North Carolina Centre for AIDS Research. ^a^Row totals may not add up to group totals due to missing data (not shown). ^b^Overall, 153 initiated PrEP at enrolment, and 25 during follow‐up. ^c^The rest belong to African, Afro‐Caribbean, Afro‐Latino or other. ^d^CESD10 score of 10+.

### 3.3 PrEP initiation and adherence

A total of 178 (79%) participants initiated PrEP during the course of the study, 153 (68%) at enrolment and 25 (11%) at a later visit (Figure 1). At the week 26 visit, based on 161 pharmacological measurements, 64% of participants who initiated PrEP met the criteria for the 90% sensitivity threshold for taking the pills ≥4 days per week in at least one of the pharmacological measures and were defined as being adherent to PrEP.

### 3.4 Correlates of PrEP initiation

In univariate analyses, participant's employment status (part‐time/self‐employed odds ratio (OR) 2.30, 95% CI 1.02 to 5.20, and full‐time OR 2.47, 95% CI 1.10 to 5.59 vs. being unemployed), perception of having enough money (OR 2.12, 95% CI 1.08 to 4.16), use of drugs other than alcohol and marijuana (OR 2.16, 95% CI 1.04 to 4.49), having any casual male partners (OR 2.11, 95% CI 1.05 to 4.27), condomless anal sex with HIV‐infected or unknown status casual male partner (OR 4.40, 95% CI 1.66 to 11.69), knowledge that an HIV‐non‐infected partner was taking PrEP before having sex (OR 2.89, 95% CI 1.46 to 5.74) and knowledge that PrEP could be used to prevent HIV infection (OR 3.69, 95 CI 3.69 to 7.50) were significantly associated with PrEP initiation.

In multivariate analyses, only condomless anal sex with an HIV‐infected or status unknown casual partner remained statistically significantly associated with PrEP initiation (AOR: 443, 95% CI: 1.68 to 11.68).

### 3.5 Correlates of PrEP adherence

In univariate analysis, participants’ age (≥25 vs. <25, OR 4.03, 95% CI 1.96 to 8.28), two‐year college or greater (OR 6.80, 95% CI 2.54 to 18.2), full‐time employment status (OR 2.62, 95% CI 1.04 to 6.61), annual income of ≥40K (OR 4.40, 95% CI 1.66 to 11.60), perception of having enough money (OR 3.59, 95% CI 1.78 to 7.24), knowledge of male partner taking PrEP before sex (OR 2.15, 95% CI 1.07 to 4.31), and knowledge of PrEP to prevent HIV infection (OR 2.48, 95% CI 1.19 to 5.18), were significantly associated with PrEP adherence at the week 26 visit (Table 2).

**Table 2:** Correlates of PrEP initiation and adherence among Black men who have sex with men enrolled in HIV prevention trials network study 073

	PrEP initiation (N = 178^a^)			PrEP adherence (N = 161^a^^,^ b)		
	PrEP initiation % (n/N)	Unadjusted OR^c^ (95% CI)	Adjusted OR^c^ (95% CI)	PrEP adherence % (n/N)	Unadjusted OR^c^ (95% CI)	Adjusted OR^c^ (95% CI)
Age						
≥25	76% (102)	0.69 (0.34, 1.39)		76% (69)	4.03 (1.96, 8.28)^d^	2.95 (1.37, 6.37)^e^
<25	84% (76)	Ref		49% (34)	Ref	
Highest education level attained						
HS or less	77% (43)	Ref		47% (18)	Ref	
Some college or vocational school	77% (72)	1.23 (0.51, 2.96)		56% (35)	1.33 (0.57, 3.11)	
Two‐year college or greater	82% (63)	0.86 (0.38, 1.96)		83% (50)	6.80 (2.54, 18.2)^d^	
Employment status						
Unemployed, disabled or other	66% (40)	Ref		56% (19)	Ref	
Part time or self‐employed	84% (67)	2.30 (1.02, 5.20)^e^	2.11 (0.84, 5.28)	56% (35)	0.90 (0.38, 2.16)	
Full time	84% (71)	2.47 (1.10, 5.59)^e^	1.48 (0.54, 4.03)	75% (49)	2.62 (1.04, 6.61)^e^	
Annual income						
<$20K	74% (80)	Ref		54% (38)	Ref	
$20K to $40K	85% (47)	1.87 (0.77, 4.57)		68% (30)	2.17 (0.92, 5.09)	
≥$40K	80% (48)	1.38 (0.57, 3.31)		73% (32)	4.40 (1.66, 11.6)^e^	
Have enough money, past six months						
Yes	85% (114)	2.12 (1.08, 4.16)^e^	1.81(0.79, 4.14)	74% (78)	3.59 (1.78, 7.24)^d^	3.58 (1.66, 7.74)^e^
No	70% (64)	Ref		45% (25)	Ref	
Health insurance						
Yes	77% (120)	0.74 (0.35, 1.55)		66% (71)	1.38 (0.68, 2.81)	
No	82% (58)	Ref		60% (32)	Ref	
Gay identity						
Yes	81% (135)	1.94 (0.95, 3.96)		66% (83)	1.94 (0.88, 4.31)	
No	72% (43)	Ref		56% (20)	Ref	
Ever incarcerated						
Yes	74% (51)	0.93 (0.46, 1.90)		56% (25)	0.68 (0.32, 1.43)	
No	81% (124)	Ref		68% (77)	Ref	
Any alcohol use, past three months						
Yes	79% (158)	1.28 (0.48, 3.39)		62% (88)	0.28 (0.08, 1.05)	
No	71% (17)	Ref		82% (14)	Ref	
Any marijuana use, past three months						
Yes	79% (87)	1.30 (0.67, 2.53)		57% (45)	0.59 (0.30, 1.16)	
No	78% (88)	Ref		71% (57)	Ref	
Any other drug use, past three months						
Yes	84% (70)	2.16 (1.04, 4.49)^e^	2.30 (0.99, 5.34)	50% (9)	0.51 (0.18, 1.43)	
No	76% (108)	Ref		66% (94)	Ref	
Poly drug use, past three months						
Yes	84% (26)	1.95 (0.68, 5.59)		38% (3)	0.28 (0.06, 1.27)	
No	78% (152)	Ref		65% (100)	Ref	
Depressive symptoms						
Yes	82% (54)	1.40 (0.66, 2.96)		51% (24)	0.44 (0.21, 0.89)	
No	77% (123)	Ref		69% (79)	Ref	
Any STI						
Yes				54% (14)	0.59 (0.25, 1.41)	
No				66% (89)	Ref	
Number of male partners, past three months median (IQR)	3 (1,5)	1.06 (0.98, 1.14)		3 (1,5)	0.99 (0.96, 1.03)	
Any primary male partners, past three months						
Yes	78% (58)	1.10 (0.54, 2.23)		62% (32)	0.92 (0.45, 1.86)	
No	79% (117)	Ref		65% (70)	Ref	
Any casual male sex partners, past three months						
Yes	83% (133)	2.11 (1.05, 4.27)^e^		65% (78)	1.07 (0.50, 2.32)	
No	69% (42)	Ref		62% (24)	Ref	
Condomless anal sex with HIV positive or unknown primary male partner						
Yes	88% (21)	2.05 (0.57, 2.28)		74% (14)	1.93 (0.65, 5.78)	
No	78% (156)	Ref		61% (89)	Ref	
Condomless anal sex with HIV positive or unknown casual male partner						
Yes	91% (72)	4.40 (1.66, 11.7)^e^	4.43 (1.68, 11.68)^e^	68% (45)	1.49 (0.68, 3.25)	
No	72% (105)	Ref		61% (58)	Ref	
Any female sex partners, past three months						
None	80% (157)	Ref		66% (96)	Ref	
At least one	70% (19)	0.55 (0.22, 1.41)		43% (6)	0.34 (0.11, 1.07)	
Knowledge of partner taking PrEP before sex						
Yes	86% (104)	2.89 (1.46, 5.74)^e^	1.68 (0.72, 3.90).	69% (68)	2.15 (1.07, 4.31)^e^	2.22 (1.03,4.79)^e^
No	70% (71)	Ref		56% (34)	Ref	
Knowledge of PrEP to prevent HIV infection						
Yes	86% (105)	3.69 (1.81, 7.50)^f^	2.14 (0.89, 5.17)	69% (68)	2.48 (1.19, 5.18)^e^	
No	69% (70)	Ref		56% (34)	Ref	
Number of C4 sessions (median, IQR)	NA	NA		5 (2, 6)	0.87 (0.72, 1.04)	

^a^Row percentages are based on non‐missing data. ^b^Includes participants who had initiated PrEP by week 26. ^c^All models were adjusted for study site. ^d^ *p* < 0.001. ^e^ *p* < 0.05. ^f^C4 is a continuous variable in the regression model.

In multivariate analysis, older age (≥25 vs. <25, OR 2.95, 95% CI 1.37 to 6.37), perception of having enough money (OR 3.58, 95% CI 1.66 to 7.74), and knowledge of male partner taking PrEP before sex (OR 2.22, 95% CI 1.03 to 4.79) were associated with an increased odds of PrEP adherence at the week 26 visit.

### 3.6 Adverse events

Twenty‐one serious adverse events (SAEs) were reported during the study, with five among participants who had not initiated PrEP. One of the 16 SAEs reported for PrEP initiators was judged to be related to the PrEP regimen (i.e. migraine equivalent syndrome). The most common SAEs were psychiatric or nervous system disorders (n = 9) and injuries (n = 4).

Reported abnormal laboratory values included 11 creatinine elevations (one grade 2 (moderate) and 10 grade 1 (mild)); nine were in participants who initiated PrEP, with six events judged to be related to the study PrEP regimen. All creatinine elevations in participants who initiated PrEP had resolved prior to their exit visit. Creatinine elevations did not result in discontinuation of study medication. Two fractures were reported for participants and were judged to be unrelated to the study medication.

Among those who initiated PrEP, no increases were reported over time in the number of male partners with median of 3 (IQR: 1 to 5) (Table 2).

### 3.7 HIV incidence

Among the 178 men who initiated PrEP, five incident HIV infections occurred in 172 person‐years (PY) of follow‐up (annualized incidence = 2.9; 95% CI: 1.2 to 7.0) compared to three infections in 39 PY of follow‐up (annualized incidence = 7.7; 95% CI: 2.5 to 24.1) among men who never initiated PrEP; the difference between the two incidence rates was non‐significant (*p *=* *0.1849). Of the five HIV seroconversion among PrEP initiators, two had permanently discontinued PrEP at 50 and 272 days prior to detection of HIV seroconversion; two participants had no detectable TFV or FTC in any samples; and one participant had a low level of FTC consistent with ≤1 dose per week indicating that the participant was not adherent to the daily regimen 64.

## 4 Discussion

HPTN 073, to the best of our knowledge, was the first open‐label study to specifically evaluate PrEP initiation, and adherence among Black MSM in the US that utilized a theoretically principled, culturally informed intervention to support PrEP uptake and adherence. Findings from this demonstration project suggest that recruitment, PrEP uptake/initiations, adherence and retention of Black MSM was feasible. Our findings on initiation and adherence to PrEP are comparable to, or exceeded, those reported in large‐scale PrEP randomized controlled trials 32, 34, but included relatively small samples of Black MSM. Based on limited safety and tolerability findings for this population, our results provide additional data 33, 41 that support the safety of daily oral PrEP among BMSM.

Our findings suggest BMSM are willing to initiate PrEP and that this decision can be supported with interventions and improved access to consistent healthcare 5, 6, 7, 8, 9, 10, 11, 12, 13, 14, 15, 16, 42.

This is particularly salient in that this population faces multiple forms of structural inequalities and studies have shown that these barriers often influence PrEP uptake and utilization among Black MSM 41, 65, 66, 67. For example, in this study, multivariate analyses show that condomless anal sex with an HIV‐infected or status unknown casual partner was significantly associated with PrEP initiation, suggesting that BMSM are able to identify their own potential risk and are willing to take steps to protect themselves. This opens the possibility that if providers are educated to work with BMSM and to ask questions in culturally and contextually relevant ways, they could assist men in making decisions and developing action steps to reduce likelihood of HIV acquisition and/or transmission. Programmes and policies could adopt such practices and institutionalize these for public health impacts.

In considering this outcome, findings from this and other studies 41 suggest that BMSM have many situational (e.g. income insufficiency) and structural barriers (e.g. incarceration and racial discriminatory experiences), that both hinder access to and discourage utilization of services. These situational and contextual factors must be incorporated into biomedical and behavioural intervention services in order to be impactful for this group. Without these important activities, biomedical interventions alone are not likely to achieve their optimal goals as these situational and contextual factors have very real and powerful impacts on the lives of many BMSM 66, 67, 68.

Beyond behavioural interventions, HPTN 073 findings provide important content for programme and policy development. Care coordination models have been shown to be successful for promoting care engagement, retention and treatment adherence among people living with HIV by helping them to address social and structural impediments that interfere with attaining health goals 69, 70, 71, 72, 73, 74. There remains a significant gap in research investigating whether care coordination approaches can produce similar gains in PrEP programme retention and PrEP adherence.

### 4.1 Limitations

The findings from this study should be interpreted in the light of several limitations: lack of a randomized control design, the small sample size; use of self‐reported sexual and drug use behaviour period of recall; and social desirability. Longer term trials, which have incidence as an endpoint are needed for BMSM in the United States.

Generalizability of findings is limited as sites reflect three regions of the US (Los Angeles, North Carolina and Washington, D.C.) using convenience sampling.

Finally, the assessment of community continuation of PrEP was outside the scope of this study.

## 5 Conclusions

Findings from the HPTN 073 study contribute to advancing knowledge about uptake and adherence with PrEP among BMSM. To engage BMSM in PrEP, programmes need to commit to assist men with navigating the social complexities in which they exist. Our findings suggest that the C4 intervention may be a useful adjunct to PrEP allowing this disproportionately affected population to garner the benefits of the latter intervention for HIV prevention.

## Competing interest

The authors declare that they have no competing interests.

## Authors’ Contributions

DPW was the Chair of the HPTN 073 Study and SDF was the Co‐Chair of the study. LW was the lead behavioural scientist and LEN was the lead implementation scientist for the study. MM, LHW and SS were the site investigators for the study in their respective cities. KHM, DPW, SDF, LW, LEN, MM, LHW and SS provided scientific leadership in the conceptualization, development and implementation of the study. GB and YQC were the Protocol Statisticians and provided statistical design, data monitoring and analysis for the study. LME supervised data management for the study. EPM and CWH provided network laboratory oversight for data collection, testing, and reporting of the pharmacologic measures and technologies for the study. KHM served as a protocol team member and provided scientific medical and health‐related expertise for the study. IK provided research, data analysis and interpretation for the study and was also a Co‐PI for her respective site. PW provided project administration support for the study. JPL provided scientific leadership in all phases of community engagement for the study. CCW served as the Chair of the HPTN Black Caucus, and along with CHO provided socio‐cultural expertise for the study. All authors contributed to the writing of the manuscript.
